# Unexpected Life‐Threatening Reactions During Surgical Management of Deep Neck Infections

**DOI:** 10.1155/crid/9079149

**Published:** 2026-02-23

**Authors:** Evyatar Yefet, Moshe Shmueli, Navot Givol, Michael Pesis

**Affiliations:** ^1^ Oral and Maxillofacial Surgery Unit, Soroka University Medical Center, Beersheba, Israel, clalit.co.il; ^2^ Faculty of Health Sciences, Ben Gurion University, Beersheba, Israel, bgu.ac.il; ^3^ Clinical Research Center, Soroka University Medical Center, Beersheba, Israel, clalit.co.il

**Keywords:** adverse reaction, deep neck infections, maxillofacial infection, povidone-iodine, surgical drainage

## Abstract

**Introduction:**

Odontogenic infections may extend into deep neck spaces, posing a serious risk of airway compromise and systemic complications. Povidone‐iodine (PVI) is frequently used intraoperatively for its broad antimicrobial activity; however, data regarding its safety in confined head and neck spaces remain scarce.

**Case Presentation:**

We describe three patients treated at Soroka University Medical Center between 2020 and 2024, whose cases were reviewed in 2024 and who developed severe complications immediately following intraoperative irrigation of deep neck spaces with undiluted PVI. One patient developed transient facial nerve palsy, another experienced extensive airway edema requiring prolonged intubation, and the third suffered a cardiac arrest during surgery that necessitated an emergency cesarean delivery. None of the patients had a known allergy to iodine or prior adverse reactions to iodinated contrast media, and all events occurred within minutes of PVI exposure.

**Discussion:**

The consistent temporal relationship between PVI irrigation and the onset of severe complications raises concern for a possible localized toxic or osmotic effect. Although the exact mechanism remains uncertain, potential explanations include osmotic tissue injury due to the hypertonicity of undiluted PVI, chemical cytotoxicity, and, less likely, hypersensitivity reactions.

**Conclusion:**

This case series highlights the potential for rare, but life‐threatening reactions following PVI irrigation in the deep fascial spaces of the neck. Caution is warranted when using antiseptic irrigants in anatomically confined regions, and diluted PVI solutions should be preferred. Further studies are needed to elucidate the underlying pathophysiology and to establish safe intraoperative irrigation protocols.

## 1. Introduction

Odontogenic infections arising from untreated dental caries or periapical pathology may extend into the deep neck spaces, where the complex fascial anatomy and proximity to vital structures can lead to life‐threatening complications [1, 2]. The progression and severity of such infections depend on multiple factors, including bacterial virulence, host immune response, and the anatomical pathways of spread. Standard management involves removal of the infection source, systemic antibiotic therapy, and surgical drainage of affected spaces, often accompanied by intraoperative irrigation to reduce bacterial load and residual debris [3, 4].

Although irrigation of the surgical site is routinely performed, procedures involving extensive or confined fascial spaces of the neck carry unique risks related to tissue edema and potential airway compromise. Povidone‐iodine (PVI) is among the most frequently used antiseptic irrigants in oral and maxillofacial surgery due to its broad antimicrobial activity and generally favorable safety profile [5]. Nevertheless, reports describing severe postoperative complications following irrigation in these deep spaces are exceedingly rare.

The objective of this article is to raise awareness of potential life‐threatening complications that may occur following surgical drainage of extensive deep neck infections, particularly those involving confined fascial spaces and possible airway compromise. Although a direct causal mechanism cannot be confirmed, several pathophysiological explanations—including osmotic tissue injury, chemical cytotoxicity, or hypersensitivity reactions—may account for the clinical findings observed in the presented patients.

## 2. Methods

This case report describes three patients who developed severe postoperative complications following surgical drainage of deep neck infections performed at the Department of Oral and Maxillofacial Surgery, Soroka University Medical Center. All patients were treated between January 2020 and December 2024.

Clinical and demographic information was retrospectively collected from electronic medical records and operative notes. For each patient, data were obtained regarding age, sex, relevant medical history, source of infection, anatomical spaces involved, surgical procedure, type and concentration of intraoperative irrigant, timing and nature of postoperative complications, management, and clinical outcome.

Formal ethical approval was not required for this report because it describes a retrospective analysis of anonymized clinical cases. The report involves no experimental intervention or deviation from standard treatment protocols, and no identifiable patient information is included. Therefore, according to institutional policy and the principles of the Declaration of Helsinki, individual informed consent and ethics committee approval were not necessary.

This case report was prepared in accordance with the CARE (CAse REport) guidelines to ensure completeness and transparency of clinical reporting.

### 2.1. Case 1

**Figure 1:**
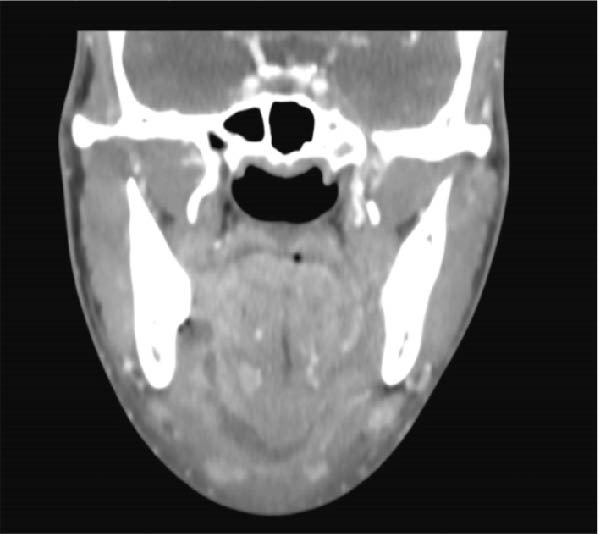
Contrast‐enhanced coronal CT of Case 1 demonstrating involvement of the right pterygomandibular, submandibular, and submental spaces.

A healthy 21‐year‐old male presented to the emergency room with severe swelling on the right side of the face and tooth pain. Clinical examination revealed stable respiratory and hemodynamic status without fever. Bilateral extraoral swelling was observed in the submandibular and submental regions, with no intraoral swelling and no limitation in mouth opening. The patient had poor dental hygiene, and the lower right third molar exhibited significant caries. Contrast‐enhanced CT imaging revealed a hypodense collection with peripheral enhancement involving the right pterygomandibular, submandibular, and submental spaces (Figure 1). The suspected origin was the right lower third molar. Under general anesthesia, the tooth was extracted, and the affected spaces were drained. Surgical irrigation with PVI was performed. Immediately after the procedure, extensive edema developed involving the submental region, bilateral neck, floor of the mouth, and tongue. The patient was sedated, intubated, and mechanically ventilated to secure the airway, and transferred to the intensive care unit (ICU). He remained intubated and ventilated for 2 weeks. Cultures revealed staphylococcus hemolyticus resistant to penicillin and clindamycin. He was treated with intravenous ceftriaxone and metronidazole, along with dexamethasone. A follow‐up CT scan showed soft tissue edema without residual abscess formation. After 2 weeks in the ICU and two additional weeks in the oral and maxillofacial surgery department, the patient was discharged with good recovery. At 1‐month follow‐up, minimal tongue mobility limitation was noted.

### 2.2. Case 2

**Figure 2:**
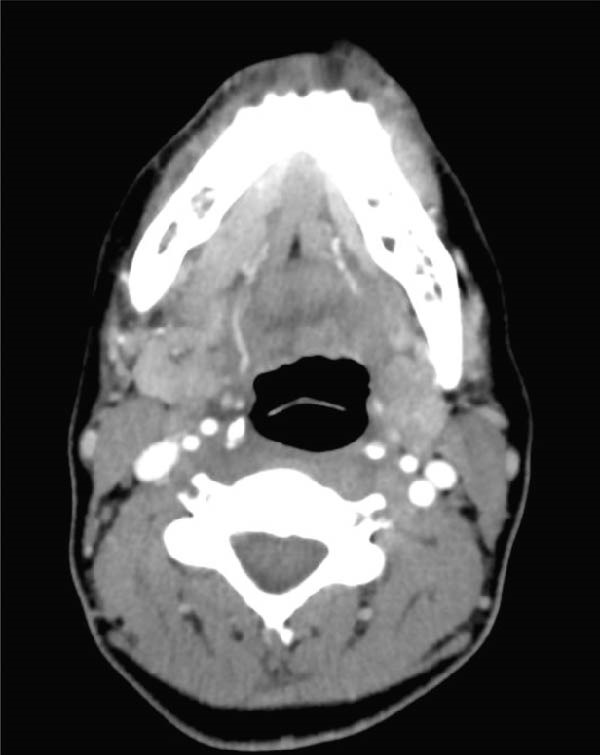
Contrast‐enhanced CT of Case 2 demonstrating a collection in the right pterygomandibular space.

A 43‐year‐old female presented to the emergency room 2 weeks after extraction of her right lower third molar. She had a known allergy to penicillin and chloramphenicol. Three days postextraction, swelling developed on the right side of her face along with trismus. She had been prescribed clindamycin 300 mg three times daily for 10 days. Clinical examination showed stable respiratory and hemodynamic parameters. Extraoral swelling was present in the right submandibular and buccal spaces. Contrast‐enhanced CT imaging revealed a hypodense collection in the right pterygomandibular space (Figure 2). Under general anesthesia, intraoral drainage and irrigation with PVI were performed. Approximately 1 h after extubation, the patient developed significant bilateral swelling of the face, neck, and chest with airway narrowing, necessitating emergency oral reintubation. A CT scan showed subcutaneous emphysema extending to the scalp, periorbital area, submandibular region, and neck. The patient was admitted to the ICU, where she was intubated and ventilated for 3 days. She received intravenous ciprofloxacin and clindamycin, along with dexamethasone. During her ICU stay, she developed complete right facial nerve palsy, trigeminal allodynia (V2 and V3 branches), and right‐sided hearing reduction. Diagnosed with facial nerve palsy, she gradually improved. After 3 days in the ICU and 7 days in the oral and maxillofacial surgery department, she was discharged. Complete recovery of facial nerve function was observed 6 months later.

### 2.3. Case 3

**Figure 3:**
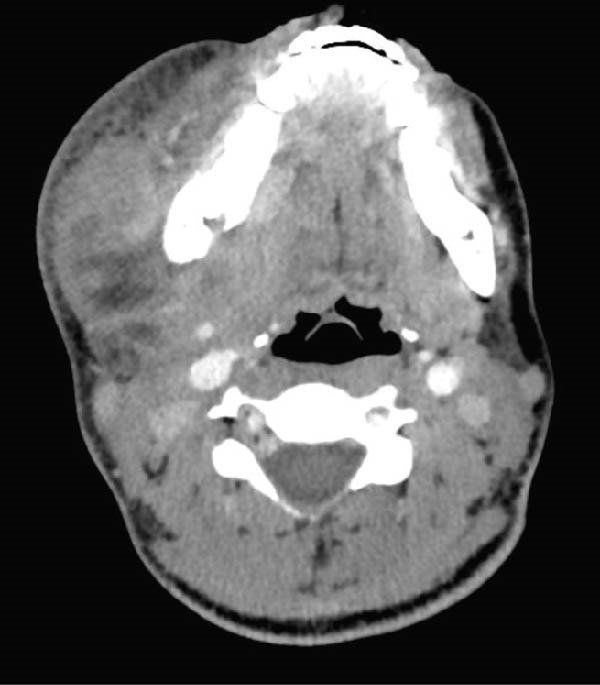
Contrast‐enhanced CT of Case 3 demonstrating multiple collections involving the submental, right pterygomandibular, right submasseteric, and right submandibular spaces.

**Figure 4:**
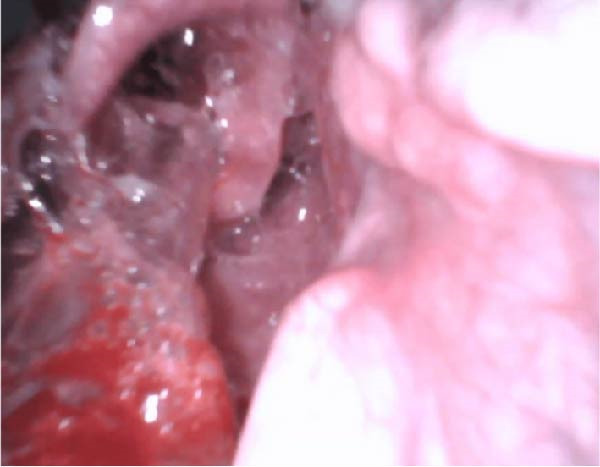
Intraoperative laryngoscopic view in Case 3 demonstrating preserved airway patency at the time of extubation.

A 22‐year‐old woman, 37 weeks and 3 days pregnant, presented to the emergency room with a 3‐week history of facial swelling and trismus. Clinical examination revealed stable respiratory and hemodynamic parameters. Fluctuant swelling was noted in the right submandibular, submental, and right buccal spaces. Intraoral examination revealed fluctuant vestibular swelling adjacent to the right lower second molar with extensive caries. Contrast‐enhanced CT imaging revealed hypodense collections in the right pterygomandibular, submasseteric, submandibular, and submental spaces (Figure 3). Under general anesthesia, surgical drainage of all affected spaces with PVI irrigation was performed. At the time of extubation, airway patency was preserved, as demonstrated in the laryngoscopy image (Figure 4). Approximately 1.5 h postextubation, right sublingual edema developed, followed by severe airway obstruction leading to cardiac arrest. Advanced cardiac life support and emergency cesarean delivery were performed. A cricothyrotomy was attempted and converted to a tracheostomy. The patient was sedated, ventilated, and treated in the ICU for 6 days. Initial antibiotics included intravenous amoxicillin‐clavulanate and dexamethasone; due to culture results identifying *Enterobacter cloacae* resistant to Augmentin and *Pseudomonas aeruginosa* sensitive to ciprofloxacin, ciprofloxacin was added. After transfer to the oral and maxillofacial surgery department for an additional 7 days, gradual improvement was observed in mouth opening, facial swelling, tongue hypesthesia, and mild right marginal mandibular branch weakness.

## 3. Discussion

Deep neck infections are known for their potential to rapidly compromise the airway, particularly when involving the deep neck spaces [1, 2]. In the present series, all patients developed acute swelling and airway obstruction immediately after irrigation of deep neck spaces with undiluted 10% PVI. This unexpected reaction occurred within minutes of irrigation and required urgent airway management.

Although PVI is widely used for its broad antimicrobial activity and low irritancy [3, 4], its application in confined cervical spaces has not been thoroughly evaluated. The acute and diffuse edema observed in these patients suggests a localized physiological or chemical reaction rather than an infectious progression.

Several mechanisms may account for this phenomenon. The first and most plausible explanation is osmotic tissue injury, as undiluted PVI is markedly hypertonic relative to physiological fluids. This hypertonicity can cause rapid transudation of plasma into the extracellular space, leading to tissue expansion and interstitial edema, particularly in the confined deep neck compartments where tissue compliance is limited [5–7]. A second possible mechanism is chemical cytotoxicity due to the oxidative activity of free iodine, which may increase vascular permeability and induce local endothelial damage [8, 9]. A third, less likely explanation involves an immediate hypersensitivity or pseudo‐allergic reaction to PVI or its excipients. All patients in this series had undergone iodine‐based contrast‐enhanced CT imaging prior to surgery without experiencing any adverse reactions, suggesting that systemic hypersensitivity to iodine is improbable. However, it is important to acknowledge the limitations in definitively characterizing the pathophysiology of these events. Due to the emergent nature of the airway compromise, no tissue histopathology, specific allergy testing, or biochemical markers of acute inflammation (e.g., serum tryptase or histamine levels) were obtained. Given the acute onset and complete resolution of symptoms, obtaining a biopsy was neither indicated nor feasible. Following recovery, patients did not consent to further allergy evaluation, and therefore the exact pathophysiological mechanism remains a diagnosis of exclusion based on temporal association.

True anaphylactic responses to PVI are exceedingly rare, with fewer than 10 documented cases, typically associated with topical, vaginal, or rectal exposure [10, 11]. Allergic reactions to elemental iodine itself are considered extremely uncommon; adverse events are more often attributed to other excipients in commercial PVI formulations [8]. Nonetheless, isolated reports have described patients developing generalized urticaria and angioedema following Betadine application, confirmed by skin prick testing [10, 11]. Considering the rapid onset, localized swelling, and anatomical confinement of the affected spaces, an osmotic mechanism related to the hypertonicity of undiluted PVI appears to be the most likely explanation for the acute edema observed in these cases.

The confined anatomy of deep neck spaces likely amplifies such reactions. Limited tissue compliance and the proximity to vital airway structures may transform a minor edematous response into a rapidly expanding obstruction. To mitigate this risk while preserving antiseptic efficacy, clinicians might consider the use of diluted PVI concentrations (e.g., 0.35%–1%). Diluted formulations are commonly used in other sensitive surgical fields, such as ophthalmology and cardiothoracic surgery, where they have demonstrated effective antimicrobial activity with significantly reduced tissue toxicity and osmotic stress [12].

## 4. Conclusions

Although PVI irrigation is widely regarded as a safe and effective adjunct in surgical wound management, this case series demonstrates that its use in the confined deep fascial spaces of the head and neck may, in rare instances, provoke severe and life‐threatening edema. The exact pathophysiological mechanism remains uncertain; however, the clinical manifestations observed necessitated immediate and aggressive airway intervention.

These findings highlight the importance of heightened vigilance during and after the use of PVI irrigation in maxillofacial and deep neck procedures. Surgeons should carefully balance the potential benefits of antiseptic irrigation against its possible risks, particularly in anatomically restricted and airway‐critical regions. Further research is needed to clarify the underlying mechanisms of these reactions and to establish evidence‐based safety guidelines for the intraoperative use of antiseptic irrigants in deep neck infections.

## Acknowledgments

The authors would like to thank the anesthesia and intensive care teams of Soroka University Medical Center for their rapid airway management and multidisciplinary collaboration during the treatment of these patients.

## Funding

The authors received no financial support for the research, authorship, or publication of this article.

## Conflicts of Interest

The authors declare no conflicts of interest.

## Data Availability

Data sharing is not applicable to this article as no datasets were generated or analyzed during the current study.
